# Machine learning modeling for the risk of acute kidney injury in inpatients receiving amikacin and etimicin

**DOI:** 10.3389/fphar.2025.1538074

**Published:** 2025-05-22

**Authors:** Pei Zhang, Qiong Chen, Jiahui Lao, Juan Shi, Jia Cao, Xiao Li, Xin Huang

**Affiliations:** ^1^ Shandong Engineering and Technology Research Center for Pediatric Drug Development, Shandong Medicine and Health Key Laboratory of Clinical Pharmacy, Department of Clinical Pharmacy, The First Affiliated Hospital of Shandong First Medical University & Shandong Provincial Qianfoshan Hospital, Jinan, China; ^2^ Department of Dermatology, The First People’s Hospital of Jinan, Jinan, China; ^3^ Center for Big Data Research in Health and Medicine, The First Affiliated Hospital of Shandong First Medical University & Shandong Provincial Qianfoshan Hospital, Jinan, China; ^4^ Department of Clinical Pharmacy, The First People’s Hospital of Jinan, Jinan, China

**Keywords:** acute kidney injury, risk factors, machine learning, amikacin, etimicin

## Abstract

**Background:**

Acute kidney injury (AKI) is a significant concern among hospitalized patients receiving aminoglycosides. Identifying the risk factors associated with aminoglycoside-induced AKI and developing machine learning models are imperative in clinical practice.

**Objective:**

This study aims to identify the risk factors associated with AKI in hospitalized patients receiving aminoglycosides, and develop machine learning models for evaluation of the AKI risk in these patients.

**Methods:**

This study retrospectively analyzed 7,028 hospitalized patients who received treatment with amikacin or etimicin between 2018 and 2020. According to the type of medication used, patients were divided into amikacin group (n = 307) and etimicin group (n = 6,901). Univariate analyses and the least absolute shrinkage and selection operator algorithm were used to screen risk factors and construct the model. The machine learning models were developed using five different algorithms, including logistic regression (LR), random forest (RF), gradient boosting machine (GBM), extreme gradient boosting model (XGBoost), and light gradient boosting machine (Light GBM).

**Results:**

The XGBoost model exhibited the most superior performance in predicting amikacin-associated AKI among the developed machine learning models. For the training set, the area under the receiver-operator characteristic curve (AUC) was 0.916, and for the test set, it was 0.841. The model can be accessed online. Regarding AKI risk in etimicin-treated patients, the GBM model demonstrated the best overall performance, with AUC values of 0.886 for the training set and 0.900 for the test set. The model was also made available online.

**Conclusion:**

These predictive models may offer a valuable tool for estimating the risk of AKI in patients receiving amikacin or etimicin, facilitating clinical decision-making and aiding in the prevention of AKI.

**Trial Registration:**

ClinicalTrials.gov NCT05533593.

## 1 Introduction

Acute kidney injury (AKI) is a common complication occurs in hospitalized patients. It is characterized by a significant decline in kidney function over a short period of time. A worldwide meta-analysis of AKI diagnoses showed that the incidence of AKI was 21.6% in adult patients and 33.6% in pediatric patients (Susantitaphong et al., 2013). The occurrence of AKI during a patient’s hospitalization not only can increase the risk of death, but also can increase the risk of readmission after discharge (Sawhney et al., 2017; Schulman et al., 2023). Previous studies have shown that patients with AKI have a mortality rate of 9.1%–23.9% (Susantitaphong et al., 2013; Brown et al., 2016) and a readmission rate of 18%–28.6% (Koulouridis et al., 2015; Silver et al., 2017).

Known major risk factors for AKI include intensive care unit (ICU) admission, shock, chronic kidney disease (CKD), hypertension, and diabetes (Zhou et al., 2019). The use of clinical drugs is also a common cause of acute kidney injury. Drug-induced acute kidney injury (D-AKI) is defined as kidney injury caused by a drug or its metabolites within 7 days after the use of one or more drugs (Mehta et al., 2015a). D-AKI accounts for 19%–40% of cases of AKI in a hospital setting (Uchino et al., 2005; Xu et al., 2015). There are many common nephrotoxic drugs which may cause D-AKI including antibiotics, diuretics, and antineoplastic drugs (Pierson-Marchandise et al., 2017; Rey et al., 2022).

Aminoglycoside drugs and their metabolites may disrupt phospholipid metabolism, which can lead to apoptosis or death of renal tubular epithelial cells (Kwiatkowska et al., 2021). Previous studies have reported several risk factors for aminoglycoside-associated AKI in elderly patients, including shock, mechanical ventilation, pneumonia, heart failure, diuretics, and vancomycin (Paquette et al., 2015; Ong et al., 2016). However, few studies focused on risk factors for AKI after treatment with aminoglycosides in adult patients. Besides, it is necessary to further explore practical tools for the early identification of AKI risk in patients, due to the lagging nature of diagnostic biomarkers. To date, few studies have developed prediction models for AKI risk in patients treated with aminoglycoside. In the present study, we aimed to identify the risk factors of AKI for two typical aminoglycosides, including amikacin and etimicin, and develop a series of machine learning models for the risk estimation of AKI in patients receiving amikacin or etimicin treatment.

## 2 Methods

### 2.1 Ethical considerations

This is a retrospective observational study conducted at the First Affiliated Hospital of Shandong First Medical University. The Institutional Review Board committees at the First Affiliated Hospital of Shandong First Medical University approved the study (No. YXLLKY-2022-024). The requirement for informed consent was granted a waiver due to the retrospective nature and minimal risk of this study.

### 2.2 Study design and participants

**FIGURE 1:**
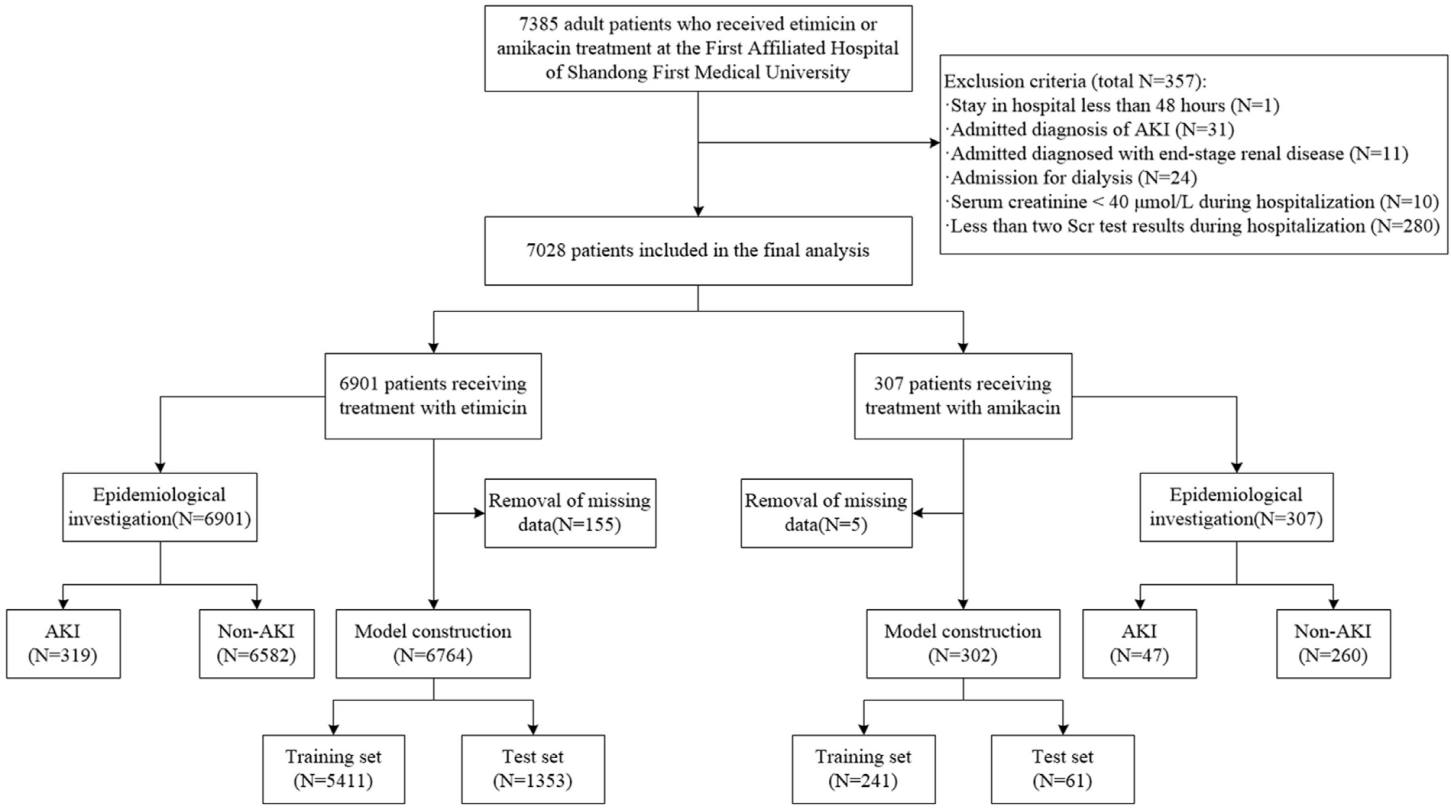
Flowchart of patient screening process.

We enrolled patients who received etimicin or amikacin treatment at the center between 1 January 2018 and 31 December 2020. Patients were excluded if they met the following criteria: (1) aged <18 years; (2) hospital stay <48 h; (3) AKI was diagnosed on admission; end-stage kidney disease, or dialysis; (4) Serum creatinine (SCr) < 40 μmol/L during hospitalization, which are not considered clinically plausible and may distort the analysis; (5) less than two SCr test results during hospitalization; (6) incomplete medical records. Initial administration date of the first aminoglycoside administered, chosen as the exposure date, was determined from electronic medical record system. Records of each hospitalization were regarded as an independent case for patients admitted to hospital more than once during the study period. The study cohort selection process is shown in Figure 1.

### 2.3 Data collection and variables

The data for this study was collected from Shandong Provincial Qianfoshan Hospital Healthcare Big Data Platform. The platform integrates multi-source data from hospital information system, electronic medical records, laboratory information management system, picture archiving and communication system, nursing information system. The encrypted personal identification number was used as a unique identifier to interlink each person’s data information in the above-mentioned database. Based on a literature review and expert opinions, we identified fifty-eight candidate predictor variables (Supplementary Material).

For each patient, only clinical and laboratory variables measured prior to the onset of AKI (or the last SCr test in non-AKI patients) were included as candidate predictors. For AKI cases, we used the most recent measurement prior to the first creatinine elevation that met the KDIGO criteria. For non-AKI patients, we selected values closest to the time of discharge or the last aminoglycoside dose. This ensured that all predictor variables temporally preceded the outcome.

In electronic medical record systems, missing data is a common occurrence. Less than 10% of the missing values were found in all variables. For missing variables, we did not perform imputation and opted for complete-case analysis. Although tree-based algorithms such as gradient boosting can internally handle missing values, we excluded records with missing data to ensure consistency in preprocessing across all model types and simplify interpretation. Furthermore, none of the dichotomous variables used in our study were missing.

### 2.4 AKI definition

The diagnosis of AKI was based on SCr changes in accordance with the 2012 Kidney Disease: Improving Global Outcomes (KDIGO) Clinical Practice Guideline for AKI (Khwaja, 2012). AKI would be diagnosed if met one of the following criteria: (1) within 48 h, the absolute value of SCr increased ≥0.3 mg/dL (26.5 μmol/L); (2) known or speculated increase of SCr within 7days ≥1.5 times of baseline value; (3) urine volume ≤0.5 mL/kg/h for more than 6 h. Due to the influence of multiple factors on urine output, it is difficult to record in retrospective studies. In the present study, we defined AKI only according to the change in SCr. The SCr baseline was defined as the last laboratory measurement 7 days prior to receiving amikacin or etimicin treatment.

Given the retrospective nature of this study, it was not possible to definitively establish a causal relationship between aminoglycoside exposure and AKI. The temporal sequence-based approach used to evaluate the potential linkage between AKI and aminoglycoside administration was defined as follows: For patients who developed AKI, drug exposure was considered to be within 72 h prior to the first observed SCr elevation meeting the KDIGO AKI criteria. For non-AKI patients, we selected variables measured prior to the last serum creatinine test performed during the hospital stay. In cases where the last SCr test was unavailable, we used the most recent measurements before the last aminoglycoside dose or discharge (whichever occurred earlier). This approach ensured that all predictor variables were measured prior to the onset of AKI (or last exposure to aminoglycosides in non-AKI patients). Patients with AKI due to other identifiable etiologies (e.g., sepsis, acute obstructive uropathy, contrast-induced nephropathy, reperfusion injury) were excluded based on explicit documentation in clinical course records. All AKI events were temporally linked to aminoglycoside exposure, and medication administration records were reviewed to confirm that drug initiation preceded AKI onset.

### 2.5 Statistical analyses

R software (version 3.6.3) was used to statistically analyze the data obtained in the study. The significance threshold for all statistical tests was 0.05, and two-sided tests were conducted for all statistical tests. The Kolmogorov-Smirnov test was initially employed to assess the normal distribution of continuous variables. Variables conforming to a normal distribution were presented as mean ± standard deviation, and group comparisons were conducted using the t-test. For variables not following a normal distribution, they were expressed as median (interquartile range, IQR), and the Wilcoxon rank sum test was employed for intergroup comparisons. Categorical variables, represented as counts (n) or percentages (n%), underwent group comparisons using the chi-square test.

### 2.6 Machine learning model development and validation

The patients included in the study were randomly divided into a training set and a test set in a ratio of 8:2. The training set data were only used for training and parameter tuning of the AKI risk prediction model, and the test set data were used to evaluate the model performance obtained from the training set.

In order to avoid overfitting of the model caused by too many variables, data dimension reduction and feature screening are carried out step by step. Firstly, univariate analysis was used to initially screen out information on variables that were significantly correlated (*P* < 0.05) with AKI. Then, the Least Absolute Shrinkage and Selection Operator (LASSO) algorithm further performed data dimensionality reduction, and the optimal penalty coefficient (λ) was determined by validation with the ten-fold crossover method to screen out the characteristic variables with predictive value. Based on the screened variables, we used five different machine learning methods to construct prediction models, including logistic regression (LR) (Jiang et al., 2020), random forest (RF) (Breiman, 2001), gradient boosting machine (GBM) (Friedman, 2001), eXtreme gradient boosting (XGBoost) (Ester et al., 2022), and light gradient boosting machine (LightGBM) (Ke et al., 2017). We also ranked the variable importance by incorporating the feature variables into the RF model. All statistical analyses and model development were performed using R software (version 3.6.3). The following R packages were utilized in this study: glmnet (version 4.1-1) for LASSO regression, randomForest (version 4.6-14), xgboost (version 1.0.0.2), gbm (version 2.1.5), and lightgbm (version 3.2.1 for machine learning model construction and evaluation, pROC for ROC curve plotting and AUC calculation, and caret for model training workflow and parameter tuning. For each machine learning model, hyperparameter tuning was performed using a grid search strategy combined with five-fold cross-validation within the training set. For the XGBoost model, we optimized max_depth, eta (learning rate), gamma, subsample, and colsample_bytree. For the Random Forest model, we tuned the number of trees (ntree) and the maximum tree depth. For the GBM model, we adjusted n.trees, interaction.depth, shrinkage, and n.minobsinnode. For the LightGBM model, hyperparameters including num_leaves, learning_rate, and min_data_in_leaf were tuned. The optimal combination of hyperparameters was selected based on the cross-validation folds in the training dataset. Model calibration was assessed by plotting calibration curves using the calibration_curve function from scikit-learn. Predicted probabilities were grouped into deciles, and the observed AKI incidence within each group was plotted against the mean predicted risk. Calibration was visually inspected for the best performed models.

The interactive online prediction tools were developed using the shiny (version 1.4.0.2) and shinydashboard (version 0.7.1) packages in R, and deployed through the ShinyApps.io platform.

We used the area under the subject operating characteristic curve (AUC), accuracy (ACC), sensitivity (SEN) and specificity (SPE) to assess models’ performance. The confusion matrix divides patients into four categories, including true positive (TP), true negative (TN), false positive (FP) and false negative (FN). The number of cases in which a positive sample was correctly predicted as positive is represented by TP. Similarly, TN denotes the count of cases where a negative sample was accurately predicted as negative. The FP stands for the number of cases where a negative sample was erroneously predicted as positive, while FN indicates the instances where a positive sample was mistakenly predicted as negative. Accuracy measures the overall correctness of the model’s predictions. Sensitivity assesses the accuracy of the model in predicting positive samples. The optimal cutoff threshold was determined based on the maximum Youden index, and calculated the corresponding sensitivity and specificity (Fluss et al., 2005). Specificity assesses the accuracy of the model in predicting negative samples. The Equations 1–4 for these metrics are as follows.

Youden index=SPE+SEN−1 (1)

$$\text{ACC}=\frac{TP+TN}{TP+TN+FP+FN}$$ (2)

$$\text{SEN}=\frac{TP}{TP+FN}$$ (3)

$$SPE=\frac{TN}{TN+FP}$$ (4)

To address the class imbalance, we employed algorithms tolerant to skewed distributions and used discrimination-focused metrics (AUC, sensitivity, Youden index) rather than accuracy alone.

## 3 Results

### 3.1 Characteristics and outcomes of patients treated with amikacin

Among 307 inpatients treated with amikacin, 47 (15.3%) were diagnosed with AKI based on the KDIGO criteria. The baseline characteristics of patients with and without AKI after receiving amikacin are summarized in Table 1. The age, gender, height, smoking history and the length of stay were found to have no significant difference between patients with or without AKI. While, patients in the AKI group had significantly higher hospital costs than those in the non-AKI group (91,289.6 yuan vs 100,058.8 yuan, *P* = 0.001).

**TABLE 1:** Characteristics of patients with amikacin.

Group	All patients (n = 307)	AKI (n = 47)	Non-AKI (n = 260)	*P* Value
Demographics				
Age (years)^*^	60 (18,96)	61 (20,96)	59.5 (18,96)	0.432
Gender (male)	208 (67.8%)	35 (74.5%)	173 (66.5%)	0.284
Smoking history	104 (33.9%)	17 (36.2%)	87 (33.5%)	0.718
Comorbidities				
Shock	17 (5.5%)	6 (12.8%)	11 (4.2%)	0.019
Hypoalbuminemia	57 (18.6%)	9 (19.2%)	48 (18.5%)	0.911
Respiratory failure	49 (16.0%)	9 (19.2%)	40 (15.4%)	0.517
Arrhythmia	31 (10.1%)	5 (10.6%)	26 (10.0%)	0.894
Myocardial infarction	14 (4.6%)	4 (8.5%)	10 (3.9%)	0.158
CHF	10 (3.3%)	4 (8.5%)	6 (2.3%)	0.028
Hepatic insufficiency	19 (6.2%)	4 (8.5%)	15 (5.8%)	0.473
Gastrointestinal hemorrhage	18 (5.9%)	5 (10.6%)	13 (5.0%)	0.130
Gastritis	9 (2.9%)	1 (2.1%)	8 (3.1%)	0.723
Hepatitis	8 (2.6%)	2 (4.3%)	6 (2.3%)	0.441
Hypertension	149 (48.5%)	23 (48.9%)	126 (48.5%)	0.952
Diabetes	62 (20.2%)	15 (31.9%)	47 (18.1%)	0.030
CHD	50 (16.3%)	10 (21.3%)	40 (15.4%)	0.314
Pneumonia	120 (39.1%)	19 (40.4%)	101 (38.9%)	0.838
Stroke	131 (42.7%)	24 (51.1%)	107 (41.2%)	0.017
Fatty liver	8 (2.6%)	1 (2.1%)	7 (2.7%)	0.691
Anemia	44 (14.3%)	12 (25.5%)	32 (12.3%)	0.017
Hypokalemia	18 (5.9%)	3 (6.4%)	15 (5.8%)	0.869
Hyponatremia	19 (6.2%)	3 (6.4%)	16 (6.2%)	0.952
Liver cirrhosis	7 (2.3%)	1 (2.1%)	6 (2.3%)	0.939
Malignancy	40 (13.0%)	5 (10.6%)	35 (13.5%)	0.597
Sepsis	3 (1.0%)	1 (2.1%)	2 (0.8%)	0.384
Gout	4 (1.3%)	0 (0.0%)	4 (1.5%)	0.392
Acidosis	9 (2.9%)	5 (10.6%)	4 (1.5%)	<0.001
CKD	6 (2.0%)	1 (2.1%)	5 (1.9%)	0.926
Medications				
BZDs	154 (50.2%)	28 (59.6%)	126 (48.5%)	0.161
NSAID	227 (73.9%)	35 (74.5%)	192 (73.8%)	0.929
PPI	257 (83.7%)	41 (87.2%)	216 (83.1%)	0.478
Statin	48 (15.6%)	9 (19.2%)	39 (15.0%)	0.471
Quinolones	169 (55.0%)	26 (55.3%)	143 (55.0%)	0.968
β-lactams	285 (92.8%)	45 (95.7%)	240 (92.3%)	0.401
Diuretic	225 (73.3%)	41 (87.2%)	184 (70.8%)	0.019
ARB	58 (18.9%)	6 (12.8%)	52 (20.0%)	0.244
ACEI	17 (5.5%)	2 (4.3%)	15 (5.8%)	0.676
H2RA	40 (13.0%)	5 (10.6%)	35 (13.5%)	0.597
Glucocorticoid	270 (87.9%)	44 (93.6%)	226 (86.9%)	0.195
CCB	108 (35.2%)	19 (40.4%)	89 (34.2%)	0.413
Tetracyclines	106 (34.5%)	24 (51.1%)	82 (31.5%)	0.010
MA	66 (21.5%)	15 (31.9%)	51 (19.6%)	0.059
Chemotherapy	19 (6.2%)	3 (6.4%)	16 (6.2%)	0.952
Procedural				
Surgeries	248 (80.8%)	41 (87.2%)	207 (79.6%)	0.222
Cardiac surgery	3 (1.0%)	2 (4.3%)	1 (0.4%)	0.013
Contrast examination	13 (4.2%)	0 (0.0%)	13 (5.0%)	0.012
ICU	51 (16.6%)	16 (34.0%)	35 (13.5%)	<0.001
Mechanical ventilation	53 (17.3%)	13 (27.7%)	40 (15.4%)	0.049
Laboratory values				
Platelets (x10^9^/L)^*^	200.5 (2,752)	153.5 (2,391)	209.5 (3,752)	0.001
RBC (x10^12^/L)^*^	3.5 (1.5,5.4)	3.2 (1.6,4.9)	3.5 (1.5,5.4)	0.048
WBC (x10^9^/L)^*^	8.9 (0.0.179.7)	9.2 (0.0,23.1)	8.8 (0.1,179.7)	0.849
SCr (mg/dL)^*^	56 (14,1061)	61.5 (14,431)	54.5 (21,1061)	0.051
TBiL (μmol/L)^*^	9.8 (1.6,398.2)	12.15 (3.2,398.2)	9.45 (1.6,254.3)	0.035
UA (μmol/L)^*^	194 (40,959.6)	205.5 (66,716)	189 (40,959.6)	0.161
β_2_-MG (mg/L)^*^	2.5 (0.8,43.2)	3.3 (0.8,43.2)	2.37 (1.0,17.2)	0.001
Cys C (mg/L)^*^	1.0 (0.5,7.3)	1.93 (0.59,7.29)	1.0 (0.5,6.5)	<0.001
Outcomes				
LOS (days)^*^	25.2 (4.1,187.1)	27.5 (4.1,187.1)	24.9 (5.4,77.0)	0.251
Hospital costs (yuan)^*^	128,215.6 (84,810.7,202,320.0)	91,289.6 (49,522.6,149,961.3)	100,058.8 (54,197.2,156,642.9)	0.001
CRRT	3 (1.0%)	2 (4.3%)	1 (0.4%)	0.013

*Two-sample Wilcoxon rank-sum test.

AKI, acute kidney injury; ACEI, angiotension converting enzyme inhibitors; ARB, angiotonin receptor blocker; BZDs, benzodiazepines; CCB, calcium channel blockers; CHD, coronary heart disease; CHF, congestive heart failure; CKD, chronic kidney disease; COPD, chronic obstructive pulmonary disease; CRRT, continuous renal replacement therapy; Cys C, cystatin C; H2RA:histamine type-2, receptor antagonist; ICU, intensive care unit; LOS, length of stay; MA, macrolides antibiotics; NSAIDs, non-steroidal anti-inflammatory drugs; PPI, proton pump inhibitors; RBC, red blood cells; SCr, serum creatinine; TBiL, total bilirubin; UA, uric acid; β2-MG, β2-microglobulin.

### 3.2 Development of machine learning models for AKI risk in patients treated with amikacin

**FIGURE 2:**
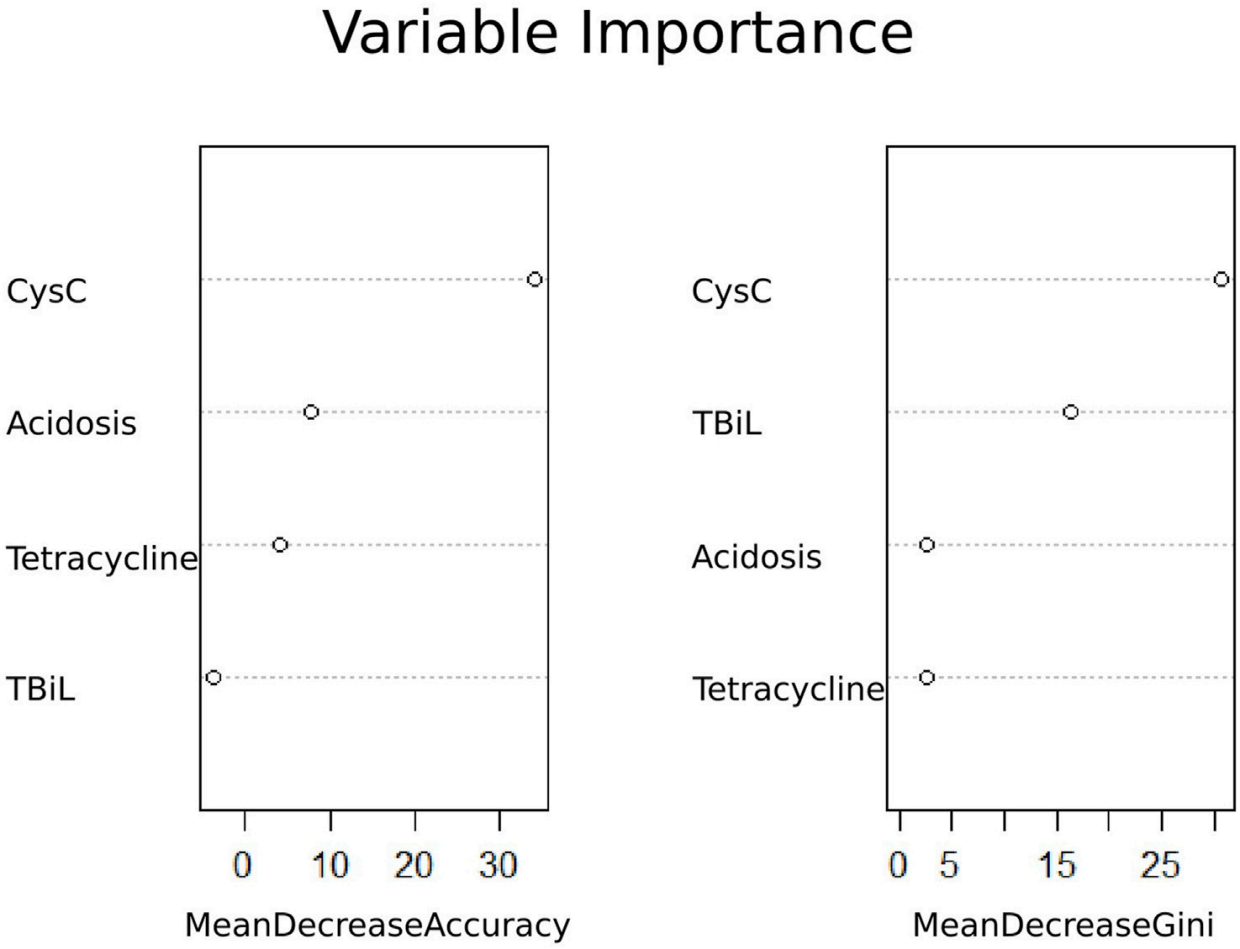
Ranking of importance of amikacin-associated AKI variables. TBiL, total bilirubin.

Based on univariate logistic regression analyses, 15 features were reduced to four potential predictors (Supplementary Figure S1). These four features included Cys C, total bilirubin, tetracycline, and acidosis (Figure 2). In addition, the feature importance plots were created by the RF algorithm to rank the importance levels. The results showed that Cys C had the greatest impact on the prediction results of amikacin-related AKI (Figure 2).

**FIGURE 3:**
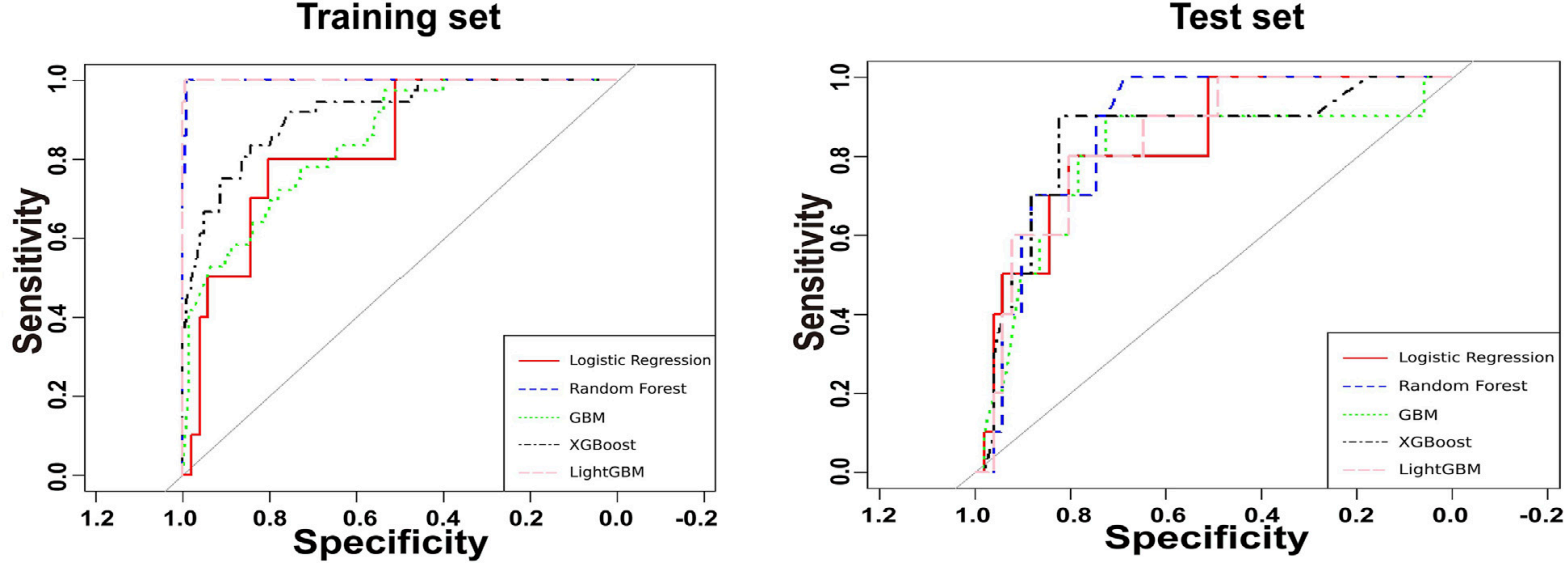
Receiver operating characteristic curves for the amikacin-AKI models. AUC, area under curve; GBM, gradient boosting machine; LightGBM, light gradient boosting machine; LR, logistic regression; RF, random forest; XGBoost, eXtreme gradient boosting.

The predictors associated with AKI screened by LASSO regression were incorporated into the machine learning model and the receiver operating characteristic curve (ROC) was plotted (Figure 3).

As shown in Table 2, the XGBoost model achieved the best ACC value and has a better balance of sensitivity and specificity. Calibration plot for the model is shown in Supplementary Figure S2. The plot displays the predicted probabilities on the x-axis and the actual observed AKI incidence on the y-axis. The calibration curve indicates good alignment between predicted probabilities and observed outcomes, suggesting that the model is well-calibrated for predicting AKI occurrence in the amikacin cohort. Therefore, we developed the web-based calculator using the XGBoost model for risk prediction of amikacin-associated AKI (Supplementary Figure S3). This online risk calculator for amikacin-associated AKI was made freely available (https://akigbm.shinyapps.io/Amikacin/). Users can enter relevant data before and predict the risk of amikacin-associated AKI on the website.

**TABLE 2:** Performance of machine learning models for amikacin-associated acute kidney injury.

Model	Youden index	Cutoff	ACC	SEN	SPE	AUC (95%CI)
Training set						
LR	0.477	0.166	0.855	0.694	0.883	0.846 (0.774–0.917)
RF	0.990	0.338	0.992	1.000	0.990	0.998 (0.995–1.000)
GBM	0.496	0.143	0.747	0.750	0.746	0.842 (0.777–0.908)
XGBoost	0.678	0.103	0.784	0.917	0.761	0.916 (0.869–0.964)
LightGBM	0.995	0.395	0.996	1.000	0.995	1.000 (0.999–1.000)
Test set						
LR	0.604	0.148	0.803	0.800	0.804	0.831 (0.702–0.960)
RF	0.686	0.087	0.738	1.000	0.686	0.867 (0.773–0.960)
GBM	0.704	0.143	0.820	0.900	0.804	0.809 (0.645–0.973)
XGBoost	0.724	0.176	0.836	0.900	0.824	0.841 (0.692–0.990)
LightGBM	0.604	0.172	0.803	0.800	0.804	0.839 (0.720–0.958)

ACC, accuracy; AUC, area under curve; GBM, gradient boosting machine; LightGBM, light gradient boosting machine; LR, logistic regression; RF, random forest; SEN, sensitivity; SPE, specificity; XGBoost, eXtreme gradient boosting; 95%CI, 95% confidence interval.

### 3.3 Characteristics and outcomes of patients treated with etimicin

Among 6,901 patients treated with etimicin, 319 (4.6%) developed AKI. The patient characteristics for those treated with etimicin are presented in Table 3. Our analysis revealed that patients in the AKI group were older and also incurred higher costs and longer hospital stays compared to those in the non-AKI group. However, there were no significant differences in gender or smoking history between patients in the AKI and non-AKI groups.

**TABLE 3:** Characteristics of patients with etimicin.

Group	All patients (n = 6,901)	AKI (n = 319)	Non- AKI (n = 6,582)	*P* Value
Demographics				
Age (years)^*^	61 (18,99)	65 (23,98)	60 (18,99)	<0.001
Gender (male)	4,600 (66.7%)	198 (62.1%)	4,402 (66.9%)	0.075
Smoking history	2,124 (30.8%)	97 (30.4%)	2027 (30.8%)	0.883
Comorbidities				
Shock	107 (1.6%)	33 (10.3%)	74 (1.1%)	<0.001
Hypoalbuminemia	248 (3.6%)	50 (15.7%)	198 (3.0%)	<0.001
Respiratory failure	265 (3.8%)	67 (21.0%)	198 (3.0%)	<0.001
Arrhythmia	327 (4.7%)	43 (13.5%)	284 (4.3%)	<0.001
Myocardial infarction	155 (2.3%)	28 (8.8%)	127 (1.9%)	<0.001
CHF	93 (1.4%)	27 (8.5%)	66 (1.0%)	<0.001
Hepatic insufficiency	95 (1.4%)	11 (3.5%)	84 (1.3%)	<0.001
Gastrointestinal hemorrhage	195 (2.8%)	16 (5.0%)	179 (2.7%)	0.016
Gastritis	1,153 (16.7%)	24 (7.5%)	1,129 (17.2%)	<0.001
Hepatitis	153 (2.2%)	13 (4.1%)	140 (2.1%)	0.021
Hypertension	2,125 (30.8%)	132 (41.4%)	1993 (30.3%)	<0.001
Diabetes	1,001 (14.5%)	76 (23.8%)	925 (14.1%)	<0.001
CHD	945 (13.7%)	79 (24.8%)	866 (13.2%)	<0.001
Pneumonia	794 (11.5%)	88 (27.6%)	706 (10.7%)	<0.001
Stroke	847 (12.3%)	86 (27.0%)	761 (11.6%)	<0.001
Fatty liver	239 (3.5%)	9 (2.8%)	230 (3.5%)	<0.001
Anemia	229 (3.3%)	32 (10.0%)	197 (3.0%)	<0.001
Hypokalemia	100 (1.5%)	15 (4.7%)	85 (1.3%)	<0.001
Hyponatremia	78 (1.1%)	13 (4.1%)	65 (1.0%)	0.015
Liver cirrhosis	148 (2.1%)	13 (4.1%)	135 (2.1%)	0.841
Malignancy	1,612 (23.4%)	76 (23.8%)	1,536 (23.3%)	<0.001
Sepsis	17 (0.3%)	7 (2.2%)	10 (0.2%)	0.02
Gout	19 (0.3%)	3 (0.9%)	16 (0.2%)	<0.001
COPD	72 (1.0%)	5 (1.6%)	67 (1.0%)	0.015
Acidosis	19 (0.3%)	8 (2.5%)	11 (0.2%)	<0.001
CKD	39 (0.6%)	5 (1.6%)	34 (0.5%)	<0.001
Medications				
BZDs	2,208 (32.0%)	138 (43.3%)	2070 (31.5%)	<0.001
NSAID	2,940 (42.6%)	217 (68.0%)	2,723 (41.4%)	<0.001
PPI	5,018 (72.7%)	261 (81.8%)	4,757 (72.3%)	<0.001
Statin	648 (9.4%)	53 (16.6%)	595 (9.0%)	<0.001
Quinolone	2,416 (35.0%)	148 (46.4%)	2,268 (34.5%)	<0.001
β-lactam	4,903 (71.1%)	272 (85.3%)	4,631 (70.4%)	<0.001
Diuretic	1951 (28.3%)	245 (76.8%)	1706 (25.9%)	<0.001
ARB	608 (8.8%)	48 (15.1%)	560 (8.5%)	0.141
ACEI	156 (2.3%)	20 (6.3%)	136 (2.1%)	<0.001
H2RA	1,055 (15.3%)	58 (18.2%)	997 (15.2%)	<0.001
Glucocorticoid	3,603 (52.2%)	246 (77.1%)	3,357 (51.0%)	<0.001
CCB	1,167 (16.9%)	84 (26.3%)	1,083 (16.5%)	<0.001
Tetracyclines	237 (3.4%)	50 (15.7%)	187 (2.8%)	0.359
MA	312 (4.5%)	25 (7.8%)	287 (4.4%)	0.075
Chemotherapy	317 (4.6%)	18 (5.6%)	299 (4.5%)	0.883
Procedural				
Surgeries	5,991 (86.8%)	276 (86.5%)	5,715 (86.8%)	0.874
Cardiac surgery	18 (0.3%)	1 (0.3%)	17 (0.3%)	0.85
Contrast examination	203 (2.9%)	21 (6.6%)	182 (2.8%)	<0.001
ICU	251 (3.6%)	82 (25.7%)	169 (2.6%)	<0.001
Mechanical ventilation	244 (3.5%)	75 (23.5%)	169 (2.6%)	<0.001
Laboratory values				
Platelets (x10^9^/L)^*^	227 (51,070)	206 (3,554)	226.5 (31,070)	<0.001
RBC (x10^12^/L)^*^	4.2 (1.2,6.9)	3.5 (1.6,5.9)	4.23 (1.2,6.9)	<0.001
WBC (x10^9^/L)^*^	7.6 (0.0.159.8)	8.8 (0.5,48.4)	7.5 (0.0.159.8)	<0.001
SCr (mg/dL)^*^	69 (18,1742)	84 (19,565)	69 (18,1742)	<0.001
TBiL (μmol/L)^*^	10.9 (1,393.3)	10.8 (2.5,360.7)	10.9 (1,393.3)	0.288
UA (μmol/L)^*^	254 (27,1030.7)	272 (57,1030.7)	253 (27,832)	0.004
β_2_-MG (mg/L)^*^	1.9 (0.8,60.8)	3.31 (1.1,60.8)	1.91 (0.8,27.9)	<0.001
Cys C (mg/L)^*^	0.9 (0.3,9.4)	1.52 (0.5,6.8)	0.84 (0.3,9.4)	<0.001
Outcomes				
LOS (days)^*^	13.0 (2.0.160.7)	17.0 (2.3,117.3)	12.8 (2.0.160.7)	<0.001
Hospital costs (yuan)^*^	66,365.7 (39,445.6,115,088.3)	33,754.3 (20,325.0,61,927.9)	34,591.4 (20,861.7,64,218.9)	<0.001
CRRT	6 (0.1%)	5 (1.6%)	1 (0.0%)	<0.001

*Two-sample Wilcoxon rank-sum test.

AKI, acute kidney injury; ACEI, angiotension converting enzyme inhibitors; ARB, angiotonin receptor blocker; BZDs, benzodiazepines; CCB, calcium channel blockers; CHD, coronary heart disease; CHF, congestive heart failure; CKD, chronic kidney disease; COPD, chronic obstructive pulmonary disease; CRRT, continuous renal replacement therapy; Cys C, cystatin C; H2RA, histamine type-2, receptor antagonist, ICU, intensive care unit, LOS; length of stay; MA, macrolides antibiotics; NSAIDs, non-steroidal anti-inflammatory drugs; PPI, proton pump inhibitors; RBC, red blood cells; SCr, serum creatinine; TBiL, total bilirubin; UA, uric acid; β2-MG, β2-microglobulin.

### 3.4 Development of a risk prediction model for AKI in patients treated with etimicin

**FIGURE 4:**
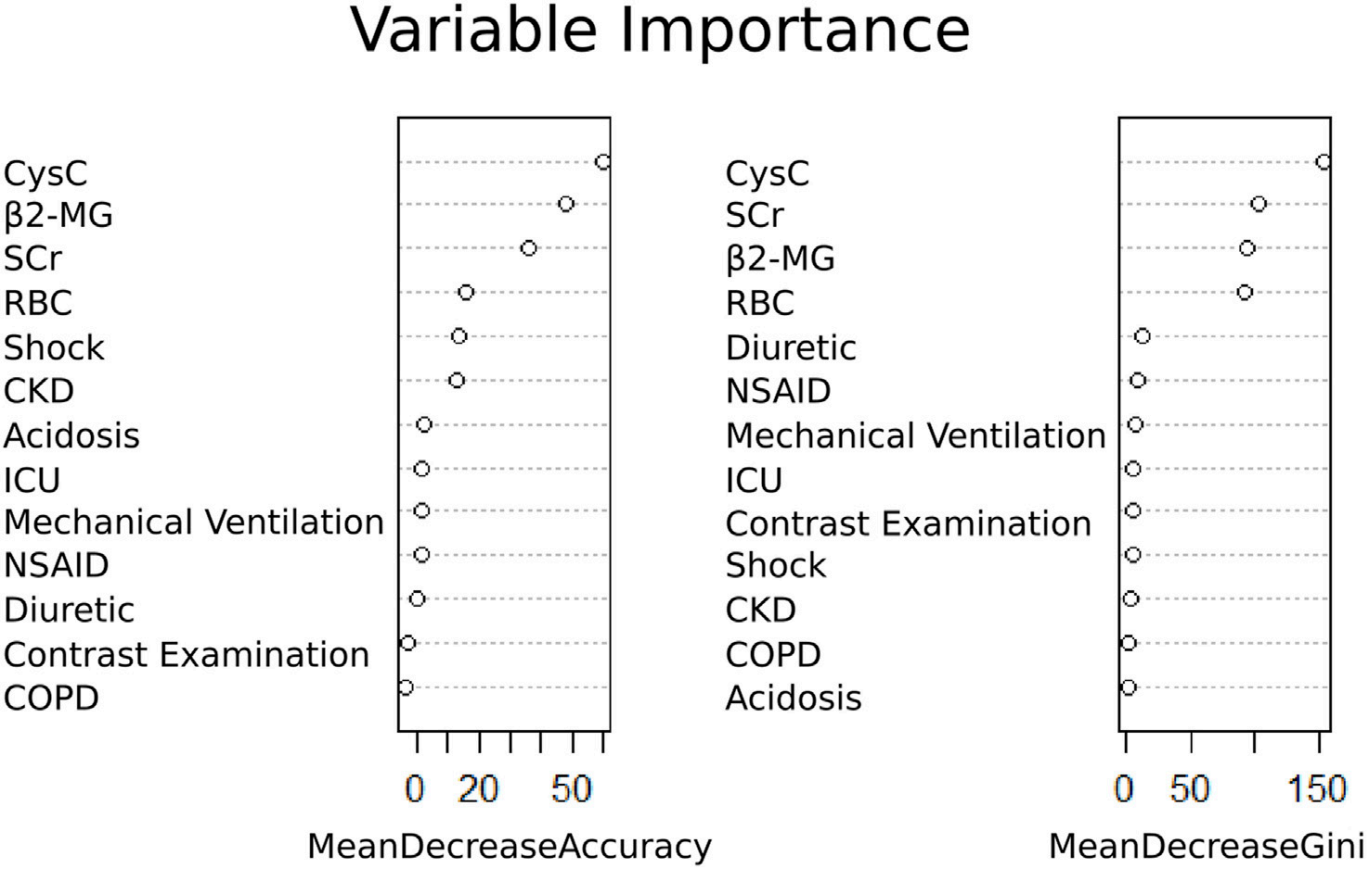
Ranking of importance of etimicin-associated AKI variables. CKD, chronic kidney disease; COPD, chronic obstructive pulmonary disease; Cys C, cystatin C; ICU, intensive care unit; NSAID, non-steroidal anti-inflammatory drugs; RBC, red blood cells; SCr, serum creatinine; β2-MG, β2-microglobulin.

Based on univariate logistic regression analysis, the 47 characteristics were reduced to 13 potential predictors (Supplementary Figure S4). These 13 features included shock, acidosis, chronic obstructive pulmonary disease, chronic kidney disease, contrast examination, admission to ICU, mechanical ventilation, combined use of diuretics or NSAIDs, RBC, SCr, β2-MG, and Cys C (Figure 4). The importance of the study variables was ranked by the RF algorithm. The results showed that Cys C, β2-MG, and SCr were the top three variables affecting the predicted outcome of etimicin-associated AKI (Figure 4).

**FIGURE 5:**
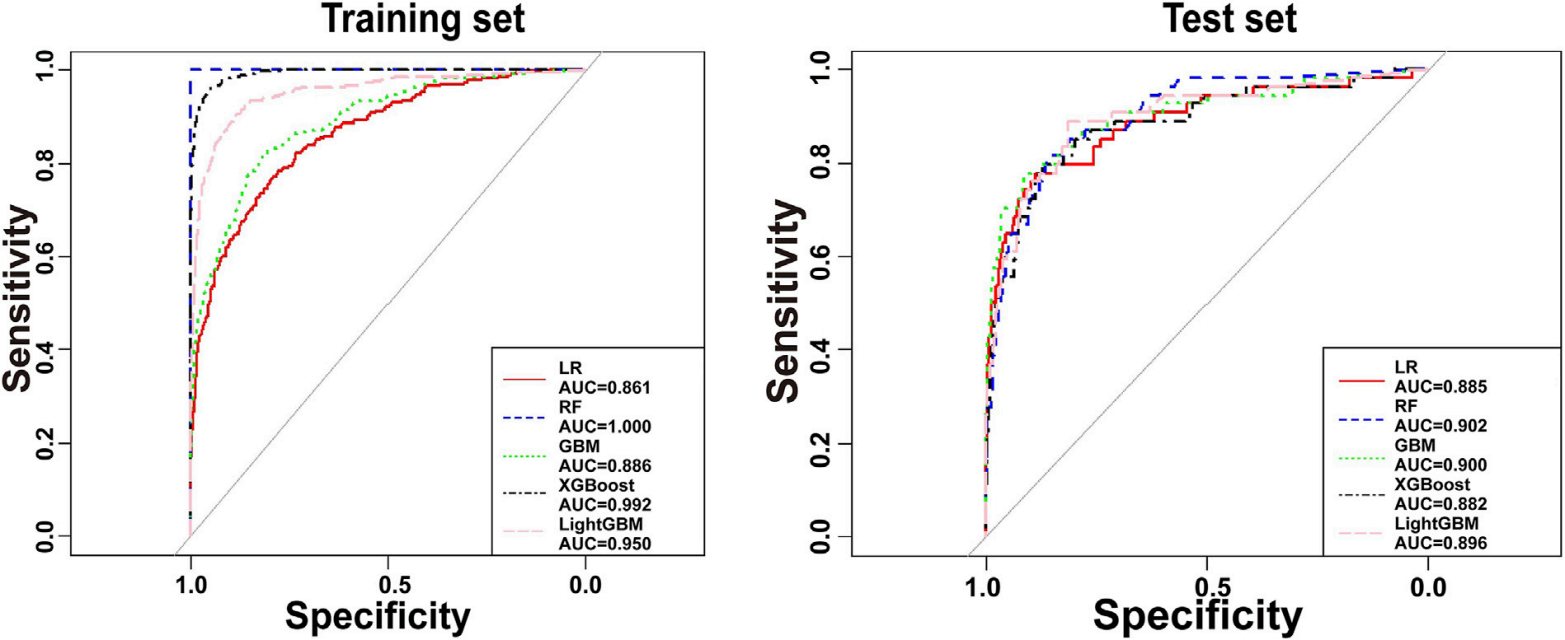
Receiver operating characteristic curves for the etimicin-AKI models. AUC, area under curve; GBM, gradient boosting machine; LightGBM, light gradient boosting machine; LR, logistic regression; RF, random forest; XGBoost, eXtreme gradient boosting.

The predictors associated with AKI screened by LASSO regression were incorporated into the machine learning model and the ROC was plotted (Figure 5).

As shown in Table 4, the GBM model preformed the best on test set. Calibration plot for the model is shown in Supplementary Figure S6. The plot illustrates the relationship between predicted probabilities and observed outcomes, with the x-axis representing predicted probabilities and the y-axis showing the actual observed AKI incidence. The calibration curve demonstrates reasonable agreement between predicted and observed values, indicating that the model is well-calibrated for predicting AKI occurrence in the etimicin cohort. We made this model available online as a web-based calculator https://akigbm.shinyapps.io/EAPP/) to predict the risk of developing etimicin-associated AKI (Supplementary Figure S7). The user enters the relevant variables on the website and clicks the Predict button, and the results are displayed in the form of high and low risks. When the results suggest that the patient has a high risk of developing AKI after receiving etimicin, it suggests that clinicians need to strengthen the monitoring of the patient’s renal function and adjust the treatment regimen in a timely manner.

**TABLE 4:** Performance of machine learning models for etimicin-associated acute kidney injury.

Model	Youden index	Cutoff	ACC	SEN	SPE	AUC (95%CI)
Training set						
LR	0.561	0.045	0.790	0.770	0.791	0.861 (0.838–0.885)
RF	1.000	0.461	1.000	1.000	1.000	1.000 (1.000–1.000)
GBM	0.634	0.036	0.818	0.816	0.818	0.886 (0.865–0.908)
XGBoost	0.910	0.102	0.949	0.962	0.948	0.992 (0.988–0.995)
LightGBM	0.788	0.066	0.888	0.900	0.888	0.950 (0.935–0.966)
Test set						
LR	0.666	0.079	0.884	0.778	0.888	0.885 (0.829–0.941)
RF	0.675	0.091	0.858	0.815	0.860	0.902 (0.861–0.944)
GBM	0.691	0.067	0.908	0.778	0.913	0.900 (0.847–0.953)
XGBoost	0.667	0.053	0.868	0.796	0.871	0.882 (0.827–0.936)
LightGBM	0.701	0.045	0.815	0.889	0.812	0.896 (0.846–0.946)

ACC, accuracy; AUC, area under curve; GBM, gradient boosting machine; LightGBM, light gradient boosting machine; LR, logistic regression; RF, random forest; SEN, sensitivity; SPE, specificity; XGBoost, eXtreme gradient boosting; 95%CI, 95% confidence interval.

## 4 Discussion

### 4.1 Incidence and analysis of AKI in patients treated with amikacin and etimicin

In our study, the incidence of AKI in inpatients treated with amikacin was 15.3% (47/307), while the incidence of AKI in inpatients treated with etimicin was 4.6% (319/6,901). The incidence of AKI in hospitalized patients taking aminoglycosides had been shown in previous studies to range from 4.3% to 26.7%, depending on the definition and study population (Selby et al., 2009; Ong et al., 2016; Ergun et al., 2022). Clinical studies have shown that etimicin has lower minimum inhibitory concentration and minimum bactericidal concentration than amikacin. Meanwhile, etimicin exhibited longer-lasting bactericidal activity based on the time kill-curve (Chaudhary et al., 2012). Yao et al. compared the nephrotoxicity of three different aminoglycosides at the same dose (Yao et al., 2020). It was shown that etimicin had lower nephrotoxicity compared to amikacin and gentamicin.

AKI patients incurred significantly higher costs and longer hospital stays than non-AKI patients. In addition, the probability of patients in AKI group to undergo continuous renal replacement therapy (CRRT) was higher, which increased the cost of hospitalization to a certain extent. A case-control study in the United States shows that the severityof acute kidney injury is significantly associated with the risk of death (Shusterman et al., 1987). Although the overall mortality rate for patients with AKI is 21% globally, the mortality rate for patients with severe AKI is as high as 42% (Mehta et al., 2015b). Oliveira et al. found that the mortality rate among ICU patients treated with aminoglycosides was significantly higher in the AKI group (44.5%) than in the non-AKI group (29.1%) (Oliveira et al., 2009). Due to the high prevalence and mortality of in-hospital AKI, early recognition and prevention are critical to patient outcomes.

### 4.2 Analysis of risk factors of aminoglycosides-associated AKI

Previous studies have identified several independent predictors associated with AKI, including shock, COPD, CHF, CKD, chronic liver disease, nephrotoxic drugs, SCr, β2-MG, ICU admission, and mechanical ventilation (Liu et al., 2019; Yue et al., 2022; Feng et al., 2023), which were confirmed in our study as well. In addition, we observed some new risk factors associated with AKI, such as acidosis, tetracyclines, red blood cell count, TBiL, and Cys C. Studies have shown that Cys C increases earlier than serum creatinine in the early stages of kidney injury and is not affected by age, sex, or race (Dharnidharka et al., 2002; Herget-Rosenthal et al., 2004). A Meta-analysis that included 3,336 patients noted that early serum Cys C could be used to predict AKI with higher predictive power than urinary Cys C (Zhang et al., 2011). The predictive ability of serum Cys C in assessing the occurrence of AKI has been demonstrated in various patients including those with traumatic brain injury, acute aortic coarctation, and those undergoing cardiac surgery (Wang et al., 2020; Wang et al., 2021; Wang et al., 2022). TBiL is an important indicator of the liver function of the body. It has been shown that TBiL concentration is closely related to aminoglycoside nephrotoxicity (Desai and Tsang, 1988). Bikrant et al. reported that when the bilirubin content was greater than 17.7 mg/dL, the risk of AKI in patients with chronic acute liver failure was 6.17 times higher than that without chronic acute liver failure (OR: 6.17, *P* = 0.011) (Lal et al., 2018). The red blood cells play an important role in maintaining normal life activities in the body as carriers of oxygen transport. In our study, patients in the AKI group had a lower number of red blood cells in their bodies prior to drug administration compared to patients in the non-AKI group. A study of liver transplant patients showed that lower red blood cell counts were associated with an increased risk of AKI (Zeng et al., 2023). This was consistent with the results obtained in this study. Many studies have found an association between red blood cell distribution width and AKI (Ramires et al., 2022; Zhu et al., 2022). As the main organ regulating systemic HCO3-concentration, the kidney plays a vital role in maintaining acid-base balance in the body. Magalhães et al. reported that acidosis promotes a decrease in glomerular filtration rate and tubular function, increases nuclear factor κB and heme oxygenase one levels, and aggravates the degree of kidney injury (Magalhaes et al., 2016). A study based on the Food and Drug Administration Adverse Event Reporting System revealed that the risk of AKI was 1.73 times higher in patients using tetracyclines than in those not using tetracyclines (Patek et al., 2020). In another study, the combined use of tetracycline and aminoglycosides triggered an inflammatory response by releasing large amounts of inflammatory factors (IL-6). Meanwhile, the combination of these two drugs also caused a significant decrease in glutathione levels and catalase activity in kidney tissue (Elgazzar et al., 2022). With patients who have multiple potential risk factors, we recommend that clinicians intensify their attention to patients in order to make timely adjustments to their treatment prescriptions.

Furthermore, our study shows that there was no significant difference in gender between the AKI and non-AKI groups. The KDIGO guideline (2012) indicates that women are more prone to developing AKI (Khwaja, 2012). However, there is still some controversy regarding the association between gender and AKI. Loutradis et al. reported that the incidence of AKI was significantly higher in male patients than in females (11.3% vs. 7.1%, *P* < 0.001). The association between men and AKI persisted after adjusting for confounders such as age, smoking history, and alcohol consumption (*P* = 0.001) (Loutradis et al., 2021). Neugarten et al. conducted a systematic review and meta-analysis of studies on aminoglycoside-related nephrotoxicity published between 1978 and 2015 (Neugarten and Golestaneh, 2016). Their study showed that male patients have a significantly higher risk of developing AKI than female patients. The differences may due to the study population.

### 4.3 Machine learning models for the prediction of AKI risk

In recent years, machine learning techniques have become increasingly prevalent in addressing medical and clinical challenges. Several studies have developed risk prediction models for drug-related AKIs, such as vancomycin and diuretics (Pan et al., 2020; Zhang et al., 2022). Pan et al. retrospectively analyzed elderly patients treated with vancomycin between January 2016 and June 2018 at a hospital in China. Univariate analysis and multivariable logistic regression analysis revealed that vancomycin trough concentration ≥20 mg/L, surgery, the Charlson Comorbidities Index ≥4 points, concomitant use of cardiotonic drug, plasma volume expander, and piperacillin/tazobactam were risk factors for vancomycin-associated AKI in elderly patients (Pan et al., 2020). The machine learning models constructed based on vancomycin-associated AKI risk factors had AUCs of 0.828 (95% CI 0.758-0.898) and 0.736 (95% CI 0.581-0.890) in the test and validation sets, respectively. In a prior study, we constructed a risk prediction model for AKI in hospitalized patients treated with diuretics (Zhang et al., 2022). There was an AUC of 0.74 (95% CI, 0.72-0.76) for the torasemide-associated AKI prediction model, and an AUC of 0.79 (95% CI, 0.77-0.80) for the furosemide-associated AKI prediction model. In the present study, the AUC values for the AKI-prediction models associated with amikacin and etimicin were 0.841 (95% CI, 0.692-0.990) and 0.900 (95% CI, 0.847-0.953), respectively. Compared to previous studies, our model has better recognition ability. Due to the varying clinical profiles associated with different drugs, aggregating them for an overall analysis may introduce bias and potentially reduce accuracy. Therefore, it is important to select the appropriate prediction model tailored to patients receiving specific medications. These prediction models can serve as valuable tools for identifying patients at high risk of AKI. While, we acknowledge the necessity of clinical judgment in determining the optimal course of action for such patients. Aligned with the principles of personalized medicine and prudent clinical practice, clinicians may consider exploring alternative treatment options or adjusting doses for patients identified as high risk by our calculator. This approach aims to maximize clinical benefits while minimizing harm to patients, thus optimizing therapeutic outcomes.

Compared with previously published machine learning models predicting AKI risk associated with other antibiotics such as vancomycin, β-lactams, or contrast agents, our models for aminoglycoside-related AKI demonstrate several unique clinical insights. First, markers such as cystatin C and total bilirubin (TBIL), which are not commonly highlighted in AKI risk prediction models for other nephrotoxic agents, were among the top-ranked predictors in our study. This may reflect the specific pharmacokinetic characteristics and renal handling mechanisms of aminoglycosides. Second, we observed that relatively moderate baseline kidney dysfunction, rather than overt chronic kidney disease, was strongly associated with D-AKI in aminoglycoside users, suggesting a unique susceptibility window. These findings underline the importance of early biomarker monitoring and individualized dose adjustment during aminoglycoside therapy. To our knowledge, this is one of the few large-scale ML-based studies focused specifically on aminoglycoside-induced AKI risk stratification, and our results may complement existing models for other antibiotic classes in guiding safer antimicrobial therapy.

### 4.4 Strengths and limitations

Our study has several strengths. First, we identified risk factors for AKI associated with etimicin and amikacin, respectively. Second, we ranked the importance of the risk factors obtained from the screening, which helps to understand the impact of single features on the prediction model. Third, we provide a reliable prediction model for assessing the risk of AKI in patients treated with amikacin or etimicin. This will help clinical doctors identify high-risk patients with D-AKI and promptly develop the best treatment strategy.

There are also limitations in our study. First, the data was retrospectively collected from the electronic medical record systems. All patients included in the study were sourced solely one medical center, which may introduce a degree of bias. The models lacked validation against other datasets. Therefore, the applicability of our prediction models needs to be further validated in larger datasets in the future. Second, the variables included in our study included laboratory indicators. If patient test data were missing, model accuracy may be reduced. A number of patients were excluded from the final cohort, primarily due to insufficient serum creatinine measurements that precluded accurate AKI classification per KDIGO criteria, or missing values in key predictor variables. It is possible that patients with sparse creatinine or cystatin C testing were healthier and less closely monitored, thus more likely to belong to the non-AKI group. This pattern suggests a potential missing-not-at-random (MNAR) mechanism, which may introduce selection bias and limit the generalizability of our findings. Future studies with prospective designs and standardized data collection will be needed to address this limitation. Third, the calibration plots revealed slight underestimation of AKI risk at higher predicted probabilities, particularly in the amikacin model. This may be due to a limited number of high-risk cases in our dataset. In future studies, calibration can be further improved using oversampling strategies or *post hoc* recalibration techniques (e.g., Platt scaling or isotonic regression) to refine risk probability estimates before clinical deployment. Finally, overfitting is a concern in several of our models, despite our efforts to mitigate it through parameter tuning. Despite the relatively small sample size in the amikacin group (n = 307, with 47 AKI cases), we developed and internally validated the model using cross-validation and independent test sets. While the number of positive cases in the validation subset was indeed limited, the model still demonstrated reasonable performance (AUC = 0.841). This model provides a preliminary, data-driven tool for identifying patients at increased risk of AKI when treated with amikacin, and should be interpreted in conjunction with clinical judgment. While this issue may not significantly impact the usability of the models or the validity of our conclusions, it warrants attention in future research. In the future, we will address overfitting by increasing sample size and conducting substantive external validation.

## 5 Conclusion

In this retrospective study, we developed and validated machine learning models to predict AKI in inpatients treated with amikacin or etimicin. The models demonstrated good discrimination and calibration performance. Key predictors included baseline renal function, electrolyte abnormalities, and concomitant nephrotoxic medications. These findings support the feasibility of leveraging machine learning for early AKI risk stratification and may inform individualized patient monitoring strategies.

## Data Availability

The datasets presented in this study can be found in online repositories. The names of the repository/repositories and accession number(s) can be found in the article/Supplementary Material.

## Glossary

ACC: Accuracy
ACEI: Angiotension converting enzyme inhibitor
AKI: Acute kidney injury
ARB: Angiotonin receptor blocker
AUC: Area under the receiver operating characteristic curve
BZD: Benzodiazepines
CCB: Calcium channel blockers
CHD: Coronary heart disease
CHF: Congestive heart failure
**CKD**: Chronic kidney disease
COPD: Chronic obstructive pulmonary disease
CRRT: Continuous renal replacement therapy
Cys C: Cystatin C
D-AKI: Drug-induced acute kidney injury
FN: False negative
FP: False positive
GBM: Gradient boosting machine
H2RA: Histamine type-2 receptor antagonist
ICU: Intensive care unit
IQR: Interquartile range
KDIGO: Kidney Disease: Improving Global Outcomes
LASSO: Least Absolute Shrinkage and Selection Operator
LightGBM: Light gradient boosting machine
LOS: Length of stay
LR: Logistic regression
MA: Macrolides antibiotics
NSAID: Non-steroidal anti-inflammatory drug
PPI: Proton pump inhibitor
RBC: Red blood cell count
RF: Random forest
ROC: Receiver operating characteristic
SCr: Serum creatinine
SEN: Sensitivity
SPE: Specificity
TBiL: Total bilirubin
TN: True negative
TP: True positive
UA: Uric acid
WBC: White blood cell count
XGBoost: eXtreme gradient boosting
β2-MG: β2-microglobulin
95%CI: 95% confidence interval
