# Supplementary figures and images for: Machine learning modeling for the risk of acute kidney injury in inpatients receiving amikacin and etimicin
Source: Front Pharmacol. 2025 May 22;16:1538074. doi: 10.3389/fphar.2025.1538074 (PMC12142076; doi:10.3389/fphar.2025.1538074)

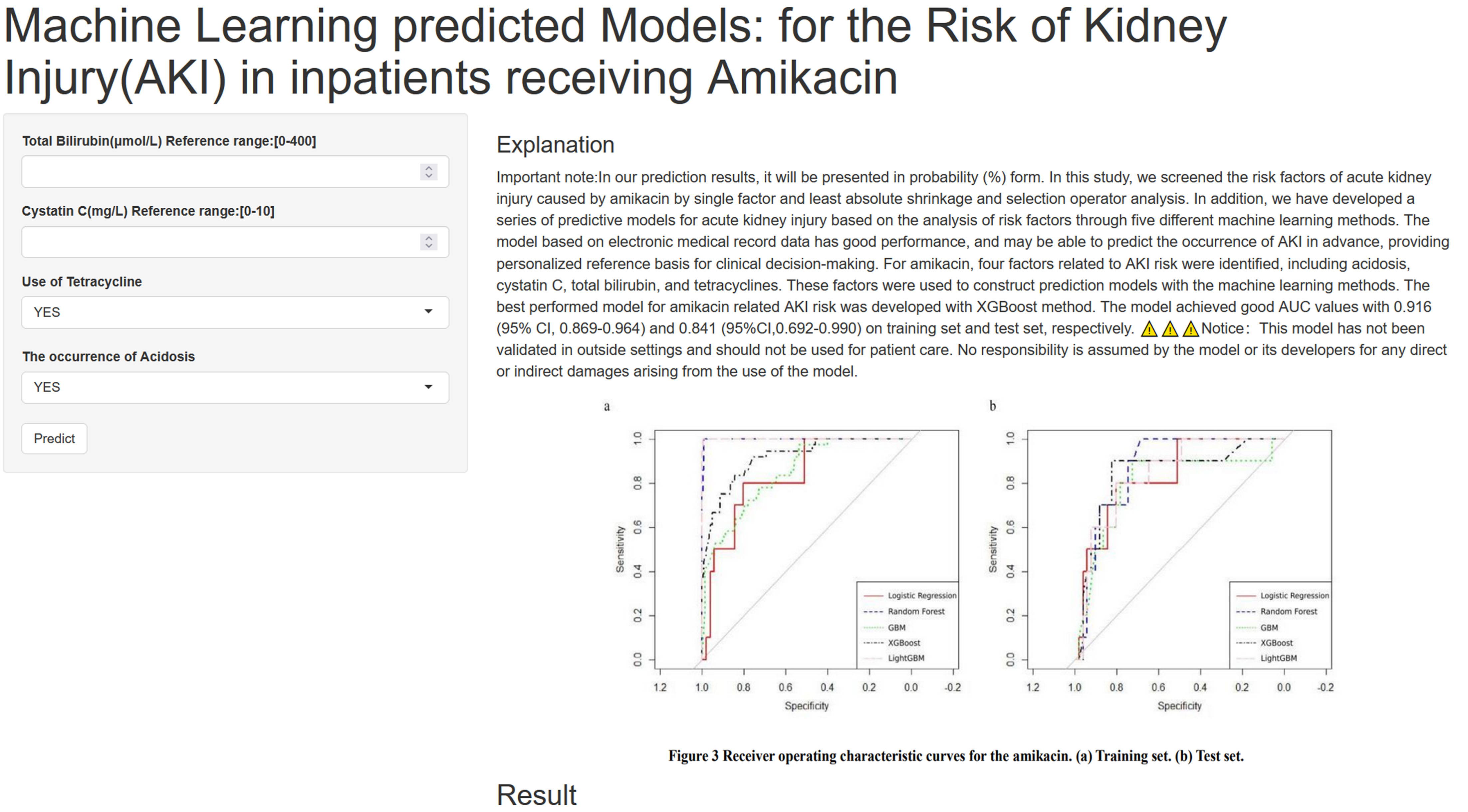

Supplement: Supplementary file 1 [file Image3.jpeg]

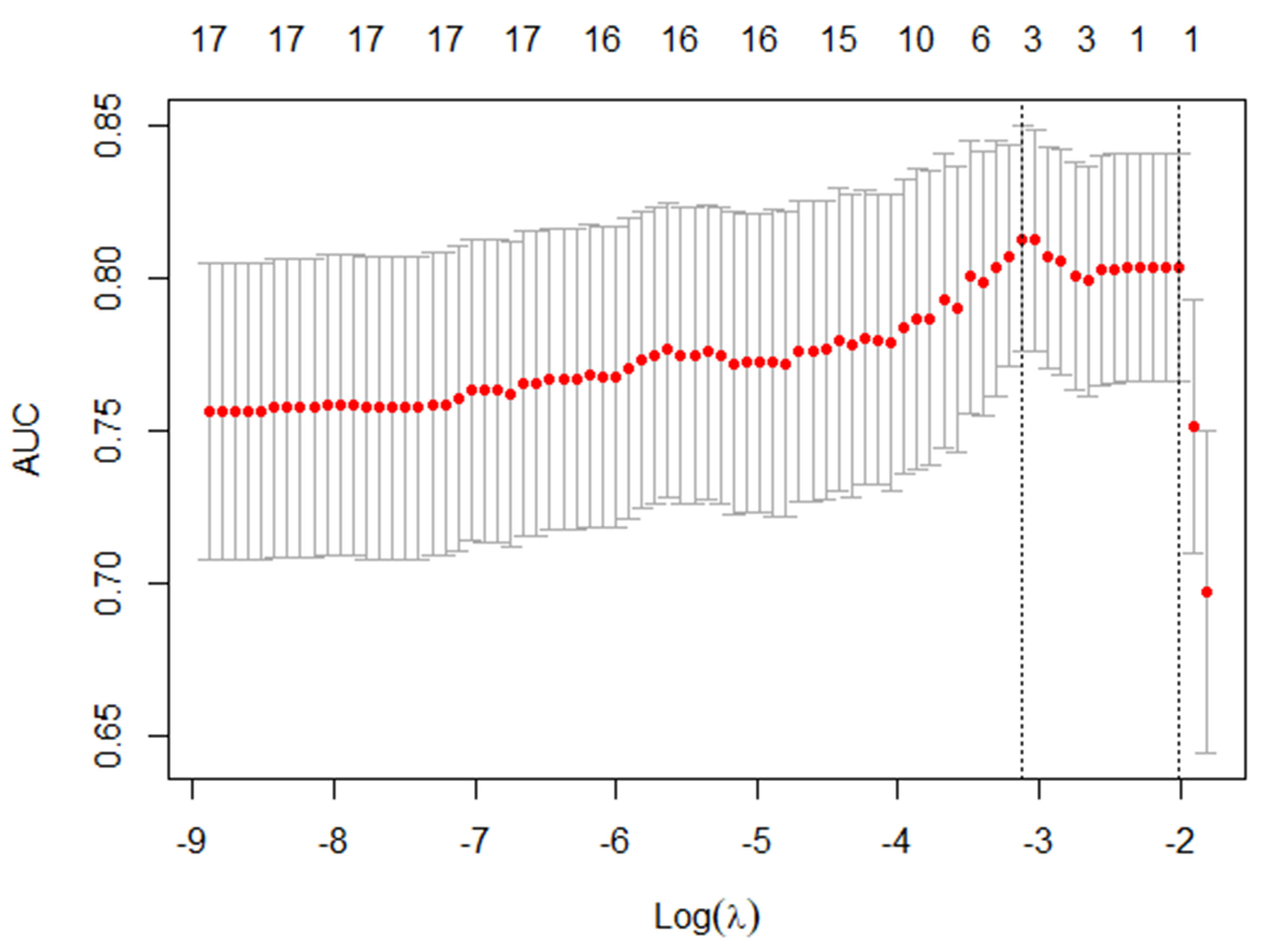

Supplement: Supplementary file 2 [file Image1.jpeg]

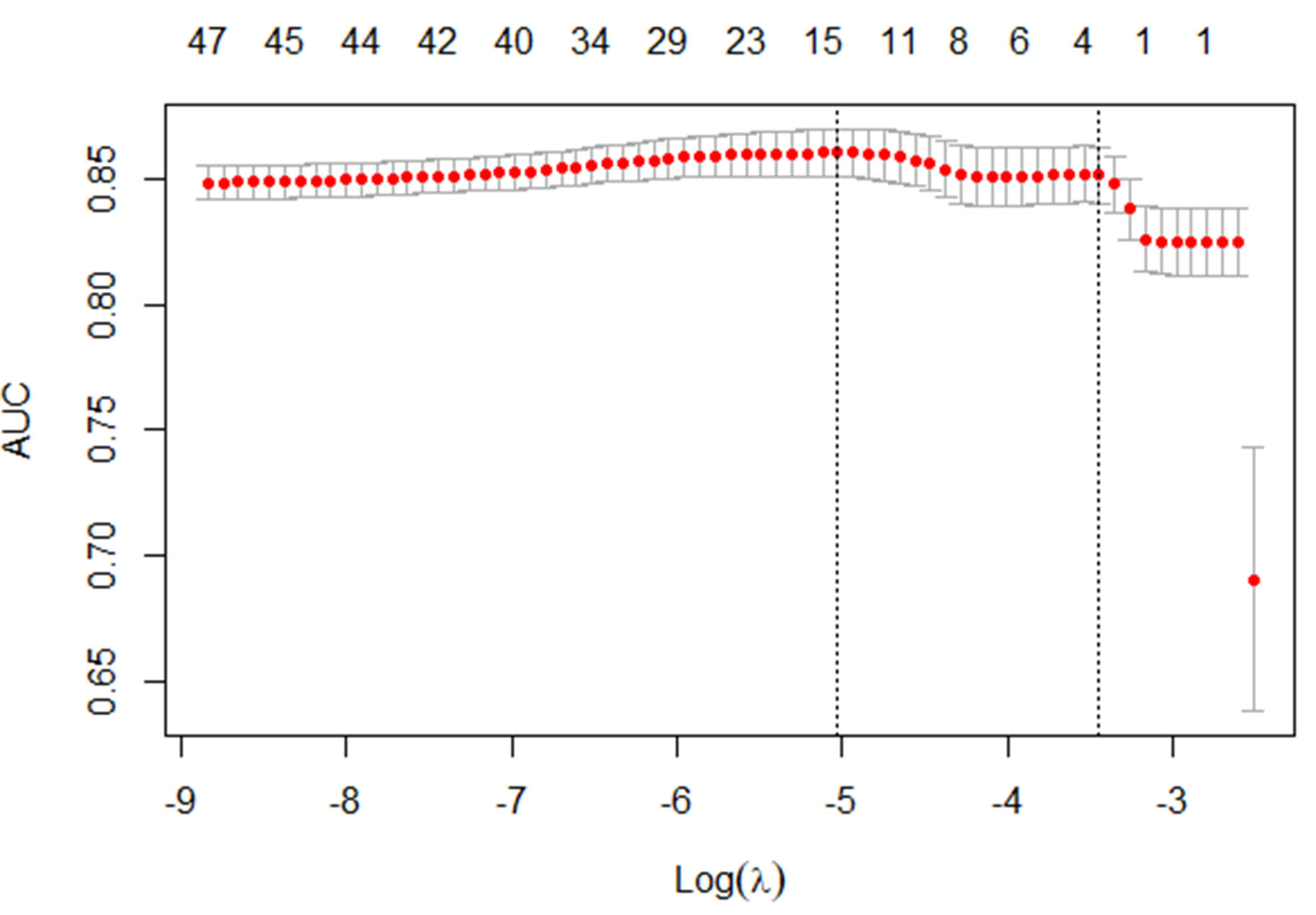

Supplement: Supplementary file 3 [file Image4.jpeg]

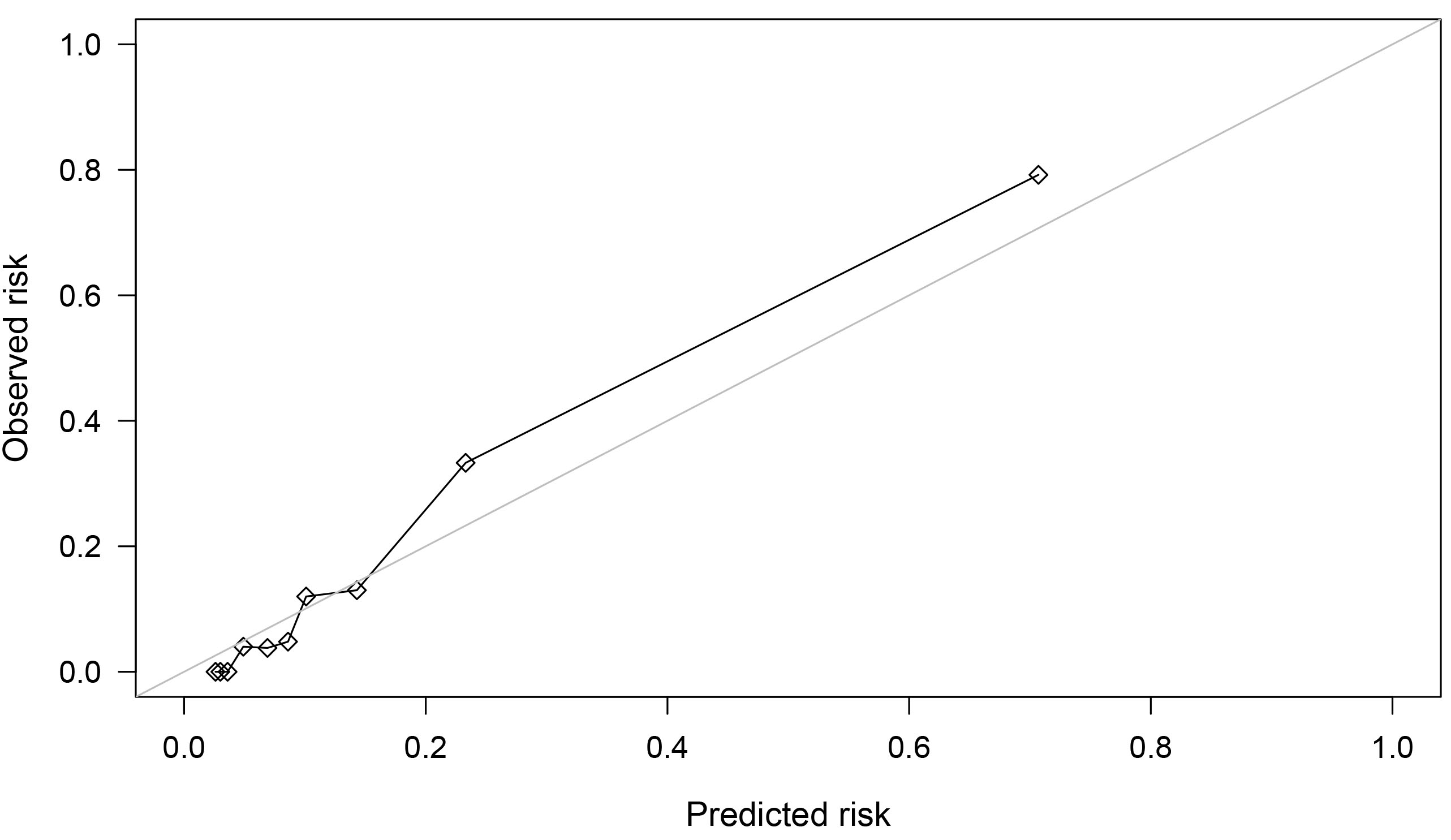

Supplement: Supplementary file 4 [file Image2.jpeg]

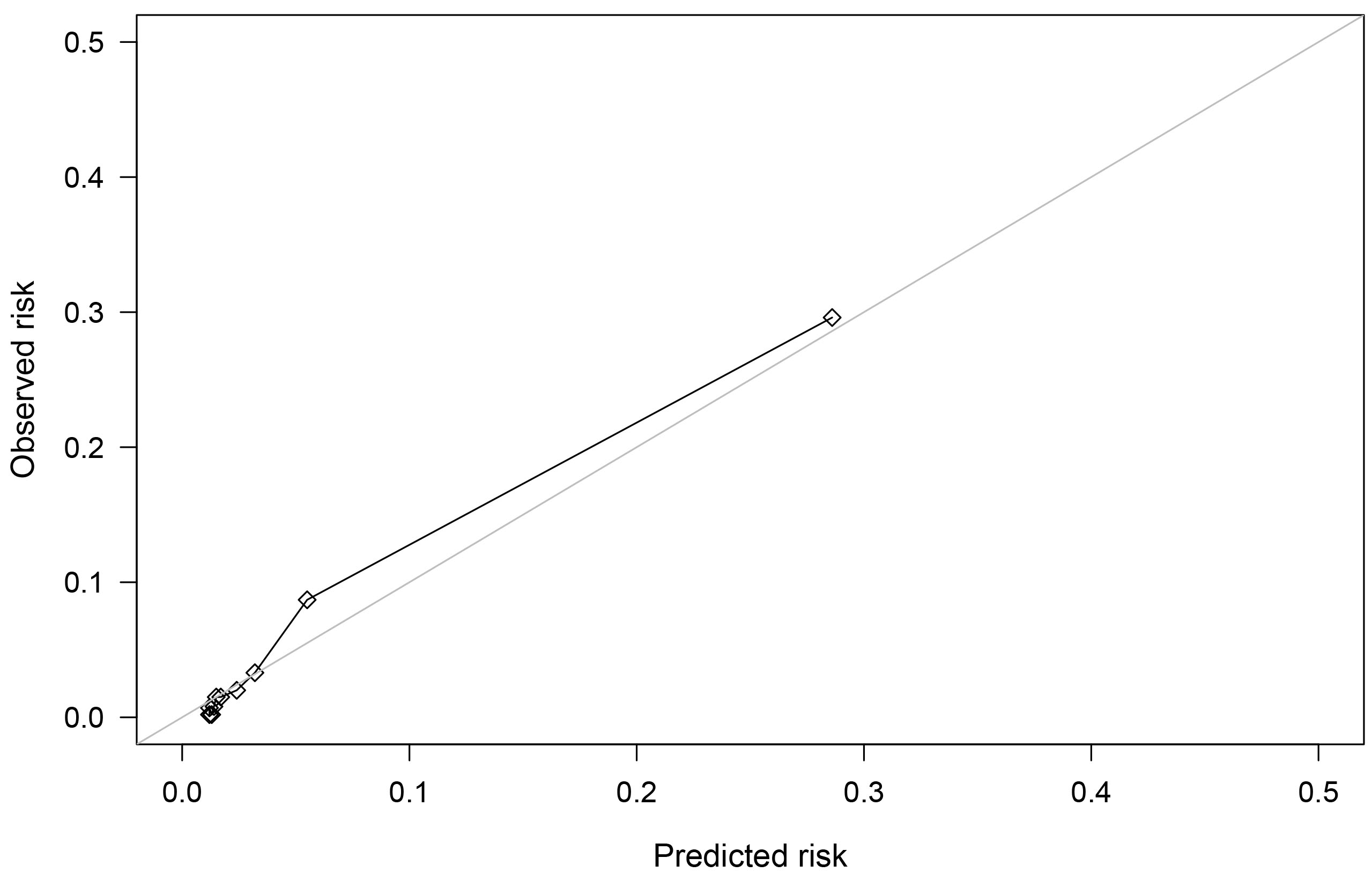

Supplement: Supplementary file 5 [file Image5.jpeg]

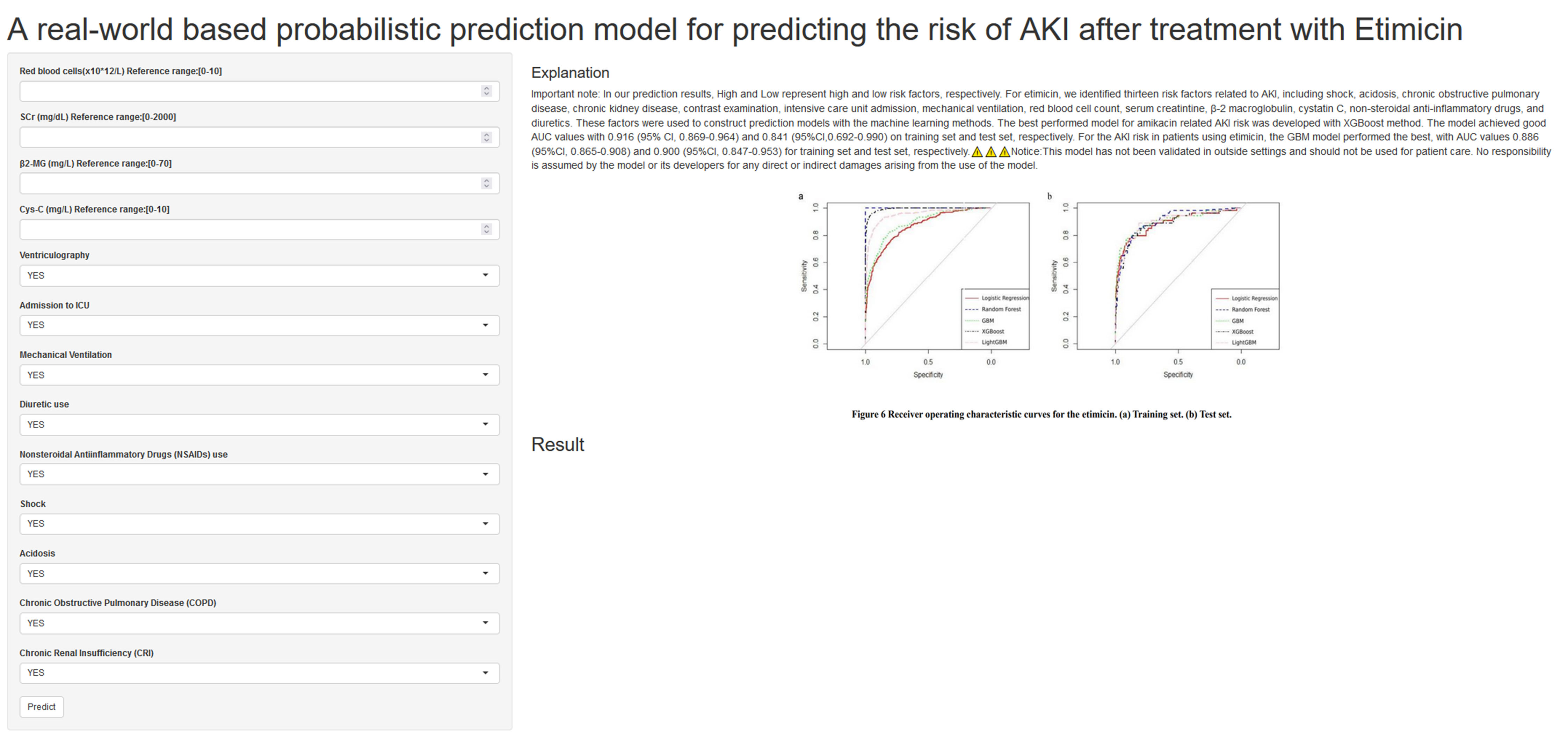

Supplement: Supplementary file 6 [file Image6.jpeg]
